# Visualization of Metastatic Lung Cancer with TiNIR

**DOI:** 10.3390/tomography9040096

**Published:** 2023-06-21

**Authors:** Seul-Ki Mun, Hyun Bo Sim, Ji Yeon Han, Hyeongyeong Kim, Dae-Han Park, Dong-Jo Chang, Sung-Tae Yee, Young-Tae Chang, Jong-Jin Kim

**Affiliations:** 1 Department of Biomedical Science, Sunchon National University, 255 Jungang-ro, Suncheon 57922, Republic of Korea; motomoto1210@naver.com (S.-K.M.); kokonun3@naver.com (H.B.S.); hanjy@scnu.ac.kr (J.Y.H.); rlaguddud78@naver.com (H.K.); carbdsinc@naver.com (D.-H.P.); 2 Department of Pharmacy, Sunchon National University, 255 Jungang-ro, Suncheon 57922, Republic of Korea; djchang@scnu.ac.kr; 3 School of Interdisciplinary Bioscience and Bioengineering, Pohang University of Science and Technology (POSTECH), Pohang 37673, Republic of Korea; 4 Department of Chemistry, Pohang University of Science and Technology (POSTECH), Pohang 37673, Republic of Korea

**Keywords:** heme oxygenase 2 (HO2), TiNIR, metastatic lung cancer, biomarkers, probe

## Abstract

The development of efficient biomarkers and probes for monitoring and treating cancer, specifically metastatic cancer, is a critical research area that can have a significant impact on both patient outcomes and drug discovery. In this context, TiNIR has been developed to detect tumor-initiating cells (TICs), with heme oxygenase 2 (HO2) as a promising therapeutic biomarker for tumor-initiating cells. In this study, TiNIR has demonstrated its effectiveness as an *in vivo* metastatic lung cancer tracker, highlighting its potential as a valuable tool in cancer research and therapy. The development of innovative approaches that selectively target metastatic cancers represents a promising avenue for improving survival rates and enhancing the quality of life of cancer patients.

## 1. Introduction

Despite decades of research and numerous approaches to treat cancer, patients still face significant challenges with this disease [1,2,3]. Even in efforts to induce apoptosis, inhibit metastasis and cancer proliferation, and regulate the immune response, the survival rates of many cancer patients remain low [4,5,6]. Metastatic cancer, in particular, poses a significant challenge due to its ability to migrate and invade other tissues, making it much more difficult to detect and treat [7,8]. Early diagnosis is crucial for successful treatment outcomes, but the plasticity of cancer presents limitations in its diagnosis due to its constantly changing nature [8,9]. Biomarkers are important tools in identifying and monitoring the progression of cancer. Therefore, it is crucial that we continue to develop and refine strategies to overcome the risks associated with cancer and its metastasis [8,9,10]. Innovative approaches such as targeted molecular imaging techniques hold promise in accurately detecting and tracking metastatic tumors [11,12]. Additionally, the development of novel biomarkers and therapies targeting specific cancer cell characteristics may provide more effective treatment options. With the development of new strategies and approaches, we may ultimately improve the survival rates and quality of life for cancer patients.

The tumor microenvironment (TME) is a complex system that comprises various heterogeneous cells, including tumor-initiating cells (TICs), differentiated tumor cells, cancer-associated fibroblasts, and tumor-infiltrated immune cells [13,14,15,16]. Despite advancements in cancer research, treating TICs, also known as cancer stem cells, is still a major challenge, as they are often resistant to conventional treatments and are responsible for tumor initiation, progression, and metastasis [17]. In an effort to address this challenge, we previously developed a selective probe called TiNIR (TIC NIR probe) to track TICs in the heterogeneous population. Furthermore, we discovered that TiNIR binds to HO2 (heme oxygenase 2), a protein highly expressed in TICs compared to differentiated cancer cells [18]. This finding provides an opportunity to selectively target TICs and develop new treatments that are more effective against these cells.

TiNIR’s near-infrared fluorescent properties make it suitable for *in vivo* applications, enabling it to reduce background interference, improve the penetration depth of the probe signal to target tissues, and increase image sensitivity [18]. We have demonstrated the usefulness of TiNIR in the isolation of TICs from bulk tumor cells and *in vivo* imaging of tumors, highlighting its potential as a valuable tool in cancer research. The development of new approaches to target TICs is important for identifying and characterizing these cells, monitoring disease progression, and evaluating the efficacy of cancer therapies. Moreover, TiNIR has the potential to be a valuable tool for investigating metastasis. Metastasis remains a significant challenge in cancer treatment, as it is responsible for most cancer-related deaths [7,9].

Clinically, surgery is usually conducted as the first step in the treatment of cancer. However, tumor recurrence and metastasis occur in many cases [4,5,6,19]. To develop an anticancer drug, subcutaneous and tail-vein-injected tumor-bearing mouse models are widely used in research. However, these models are far different from realistic treatment and make it difficult to study metastasis in cancer. Therefore, an appropriate animal cancer model is necessary [20]. A549 is a non-small-cell lung cancer (NSCLC) cell line commonly used in research related to lung cancer. A549 cells have been extensively studied and are widely used as a model system for lung cancer research [21]. Here, we established a realistic metastatic lung cancer mouse model induced by tumor surgery using the A549 cell line.

In this study, we present two potential applications of TiNIR and its biomarker HO2 in lung cancer metastasis. Firstly, we found that the metastatic cancer cell population exhibited a high level of HO2 expression both in vitro and *in vivo*, suggesting that HO2 may serve as a biomarker for metastatic cancer cells. Building on this finding, we demonstrated the selectivity of TiNIR by visualizing metastatic tumors. Secondly, the systems we employed, including the metastatic lung cancer model and assessment strategy, hold promise as a platform for discovering novel drugs aimed at combating metastatic cancer.

## 2. Materials and Methods

### 2.1. Cell Cultures

Human lung carcinoma cells (A549, KCLB 10185) were acquired from the Korean cell Line Banks (Seoul, Republic of Korea). The cells were cultured in the presence of 10% FBS (fetal bovine serum, Hyclone, Novato, CA, USA), 2-ME (2-Mercaptoethanol, Thermo, Waltham, MA, USA), HEPES (Thermo, Waltham, MA, USA), sodium pyruvate, and antibiotic–antimycotic RPMI-1640 medium (Hyclone, Novato, CA, USA). The cultures were maintained at 37 °C and 5% CO_2_. The cells were subcultured 24 h before use in the experiments.

### 2.2. Quantitative Real-Time PCR

The total RNA from the cell lines was purified using an RNeasy Mini kit (Qiagen, Valencia, CA, USA). The RNA was eluted in RNase-free water, quantified using a Nanodrop-2000 (Thermo, Wilmingto, DE, USA) and diluted 300 ng/3.5 μL in RNase-free water. Real-time PCR using a RNA-direct™ SYBR^®^ Green Realtime PCR Master Mix kit was performed according to the manufacturer’s instructions with modification. The q-PCR primers are as follows: GAPDH (FW: GTCTCCTCTGACTTCAACAGCG, RW: ACCACCCTGTTGCTGTAGCCAA), HO2 (FW: GGACAGGCGACAGCGAC, RW: CCGAGAGGTCAGCCATTCTC), vimentin (FW: AGG CAA AGC AGG AGT CCA CTG A, RW: ATC TGG CGT TCC AGG GAC TCA T), snail (FW: TGC CCT CAA GAT GCA CAT CCG A, RW: GGG ACA GGA GA AGGG CTT CTC), slug (FW: ATC TGC GGC AAG GCG TTT TCC A, RW: GAG CCC TCA GAT TTG ACC TGT C), and N-cadherin (FW: CCT CCA GAG TTT ACT GCC ATG AC, RW: GTA GGA TCT CCG CCA CTG ATT C). GAPDH was used for the housekeeping genes, and the target gene expression was quantified by a Real-Time PCR Detector (CFX Connect, Bio-rad, Hercules, CA, USA). The data were analyzed using the Δct power method.

### 2.3. Cell Image

The A549 cells were seeded in a 24-well plate (1 × 10^6^/well) and subcultured for 48 h. Subsequently, the cells were scratched by micro tips and cultured for 24 h. For imaging, the cells were maintained at 37 °C supplemented with 5% CO_2_ in the microscope. All images were quantified by EVOS 7000 and acquired with a ×4 air objective.

### 2.4. Flow Cytometry

The A549 cells were seeded in 24-well plate (1 × 10^6^/well) and sub-cultured for 48 h. Subsequently, the cells were scratched and cultured for 24 h. The cells were stained by TiNIR (100 nM, ex: 805 nm, em: 825 nm, Merak, Germany, and Senprobe, Republic of Korea) for 30 min. After washing by PBS, the cells were destained by media for 1 h. After destaining, the cells were re-suspended in PBS. TiNIR signals (channel: APC-Cy7) were detected by flow cytometry (FACS canto II, BD biosciences, San Jose, CA, USA). Data analysis was performed using FlowJo software ver 10.8.1 (TreeStar, Woodburn, OR, USA).

### 2.5. Metastatic Mouse Model

The animal study was performed under the approval of the Sunchon National University Institutional Animal Care and Use Committee (SCNU IACUC-2023-4). BALB/c nude mice (6–8 weeks, CAnN. Cg-Foxninu/CrlOri, *n* = 3 per group) were purchased from Orient-bio (Seongnam, Republic of Korea). The animals were housed in a controlled environment (22 ± 2 °C and humidity 50 ± 5%) in polycarbonate cages and fed a standard animal diet with water. To establish metastatic tumor models, A549 cells (2 × 10^6^/100 μL) were subcutaneously (S.C.) injected into the flank site of BALB/c nude mice. Approximately 8 weeks after post-injection of the A549 cells, tumors were surgically removed under anesthesia and maintained for 12–13 weeks. The mice were injected with TiNIR (100 μM in PBS, 100 μL/10 g) into their tail vein one day before ex vivo imaging. The TiNIR signal was detected on the infrared (IR) long channel (810–850 nm) using Amersham™ Image Quant 800 (GE Healthcare, Bloomington, IL, USA). This signal was analyzed using Image J software ver 1.53 (National Institutes of Health, Bethesda, Maryland, USA).

### 2.6. Tissue Imaging

The harvested lungs were washed with PBS to remove blood and frozen under the OCT (optimal cutting temperature, Surgipath FSC 22, Leica, Wetzlar, Germany). Cryosectioned tissue (14 μm) was prepared using a freezing microtome (Amos Scientific, Melbourne, Australia). The section was washed with PBS to remove OCT. The section was stained using an H&E stain kit (hematoxylin and eosin, Abcam, Waltham, MA, USA) according to the manufacturer’s instructions. The fluorescence image was stained using diamond antifade mount with DAPI (Thermo, Carthage, MO, USA). For immunofluorescence, a section was fixed within 4% formalin–PBS and incubated for 30 min at RT with 0.2% Triton–PBS. The section was incubated for 30 min at room temperature with primary antibodies (anti-HO2, 2 μg/mL, Invitrogen, Paisley, UK). After washing with PBS, sections were incubated for 30 min at room temperature with secondary antibodies (AlexaFluor488 conjugated anti-goat IgG, 1 μg/mL, Invitrogen, Paisley, UK). The image was obtained by EVOS 7000, ×20, and ×40 air objectives. The image was analyzed using Image J and Celleste image analysis software ver. 5.0 (Thermo, Carthage, MO, USA).

### 2.7. Statistical Analyses

The statistical differences between the groups were analyzed by one-way SPSS version 22 (SPSS, Chicago, IL, USA) followed by Student’s *t*-test. All values are expressed as mean ± standard deviation (SD).

## 3. Results

### 3.1. In Vitro Confirmation of HO2 Expression Levels in Actively Migrating Cancer Cells

**Figure 1:**
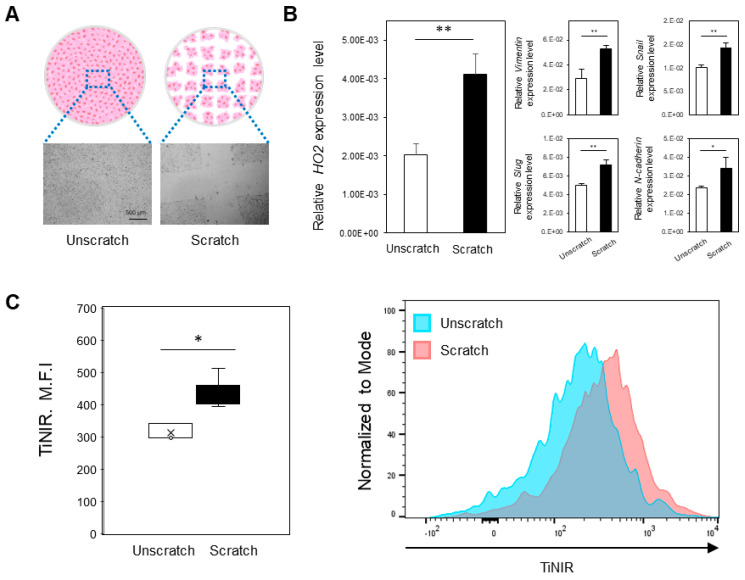
Expression of HO2 mRNA in the different culture conditions of A549 cells. (**A**) Representative cell microscope image (×4) of the experimental concept. The cells were scratched to check the expression pattern of HO2 in the migratory cancer cells. (**B**) HO2 and migration marker mRNA levels were confirmed by qPCR. (**C**) TiNIR staining pattern in the migrating cancer cell. Each cell was stained by TiNIR (100 nM, 37 °C) for 30 min and destained with PBS for 2 h. Fluorescence intensity was measured by flow cytometry. Data are presented as the mean ± SD from three independent experiments. (*p*-values were determined by *t*-test, *p* * < 0.05, *p* ** < 0.01 versus the unscratched cells).

To investigate HO2 expression during different stages of cell culture conditions, we utilized scratched cell culture conditions, which simulate the process of cell migration (Figure 1A). First, we investigated the expression level of vimentin, snail, slug, and N-cadherin to confirm the metastatic characteristics and observed up-regulation of those genes. We then measured the expression level of HO2 mRNA in both scratched and unscratched cells. Our results demonstrate that dynamically migrating cells exhibit significantly higher levels of HO2 expression compared to unscratched stationary cells (Figure 1B). To check the staining pattern of those cells, we treated them with TiNIR (100 nM) and confirmed a strong TiNIR signal from actively migrating cells under the scratched condition, compared to unscratched cells, using flow cytometry (Figure 1C). TiNIR can potentially exploit this difference in HO2 expression levels to detect metastatic cancer cells.

### 3.2. In Vivo Metastatic Mouse Cancer Model and Expression of HO2

**Figure 2:**
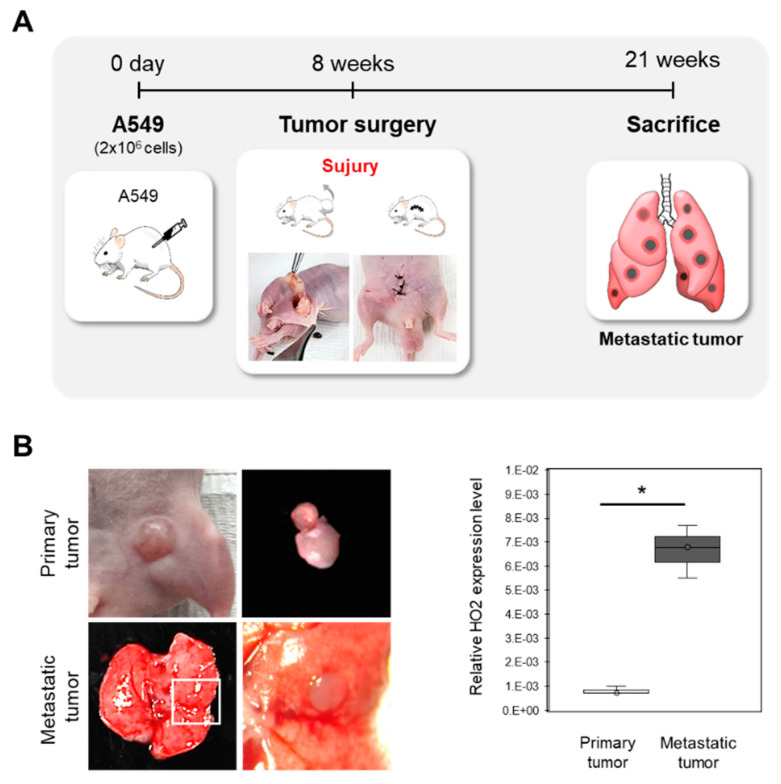
Establishment of the metastatic lung cancer model using A549 cells. (**A**) The schematic procedure of the *in vivo* experiment for the metastatic lung tumor model. (**B**) Confirmation of generating the metastatic tumor and HO2 expression. We observed metastatic tumor generation in the lung (upper) 13 weeks later from the primary tumor (bottom) surgery. The HO2 expression level of each primary and metastatic tumor was measured by qPCR. Data are shown as the mean ± SD (*n* = 3, *p*-values were determined by Student’s *t*-test * *p* < 0.05).

To establish models with a similar clinical presentation to metastasis patients, we established a metastatic lung cancer model using A549 cells implanted subcutaneously in nude mice, followed by surgical removal of the resulting tumor chunk under anesthesia (Figure 2A). This approach allowed us to create a mouse model that closely mimics the clinical presentation of metastatic lung cancer. After 12–13 weeks, we observed metastatic tumors in the lungs of the mice, confirming the validity of our model. To evaluate the potential of HO2 as a biomarker for metastatic cancer, we examined the expression levels of HO2 in both the subcutaneous primary tumors and the lung metastatic tumors. Our findings showed that the expression of HO2 was significantly higher in the metastatic tumors (6.8-fold) compared to the primary tumors, consistent with our in vitro results (Figure 2B). These results suggest that HO2 may serve as a therapeutic biomarker for metastatic cancer and that HO2 inhibitors could be a potential treatment option.

### 3.3. Visualization of Metastatic Lung Tumors by TiNIR Ex Vivo

**Figure 3:**
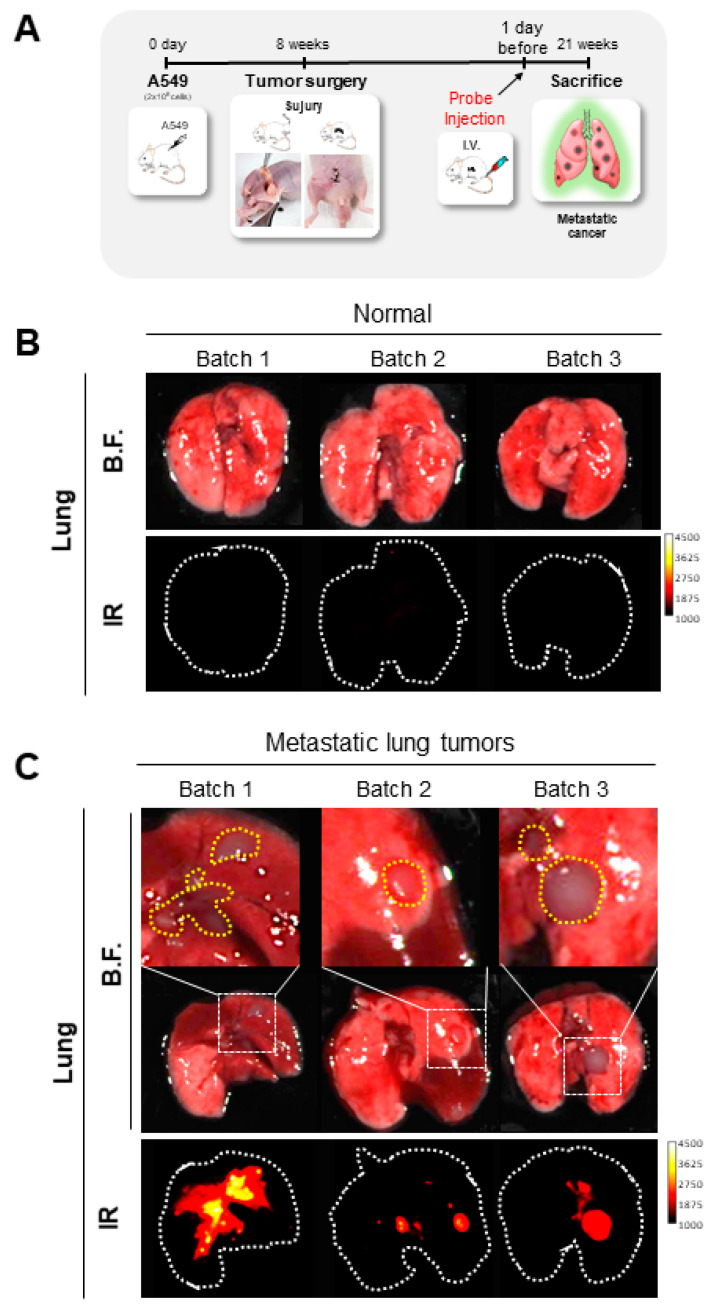
Ex vivo tracking metastatic lung tumors. (**A**) The schematic procedure of the experiment for tracking metastatic lung tumors by TiNIR. TiNIR was injected into the tail vein of the normal group and the metastatic cancer mouse model (100 μM, 100 μL/10 g) 1 day before sacrifice. TiNIR signals were detected on the infrared (IR) long channel (810–850 nm) from (**B**) the normal lung group and (**C**) the metastatic lung tumor group.

The applicability of targeted molecular imaging techniques has been emerging as a promising approach for detecting and tracking metastatic tumors. To track metastatic tumors, TiNIR (100 µM, 100 μL/10 g) was injected into the tail vein one day prior to sacrifice, and TiNIR signals were detected in the ex vivo lung tissues (Figure 3A). In fluorescence imaging, the normal lung lobes of the control mouse did not show a TiNIR signal (Figure 3B). Contrastively, the metastatic-cancer-induced mouse showed a strong and clear TiNIR signal in the tumor area (Figure 3C). Overall, the results of this study provide value for the use of TiNIR for the detection and tracking of metastatic cancer and highlight the potential of HO2 as a therapeutic target for this disease. Further research in this area has the potential to lead to the development of new diagnostic and therapeutic approaches that could significantly improve the prognosis of patients with metastatic cancer.

### 3.4. Histological Analysis

**Figure 4:**
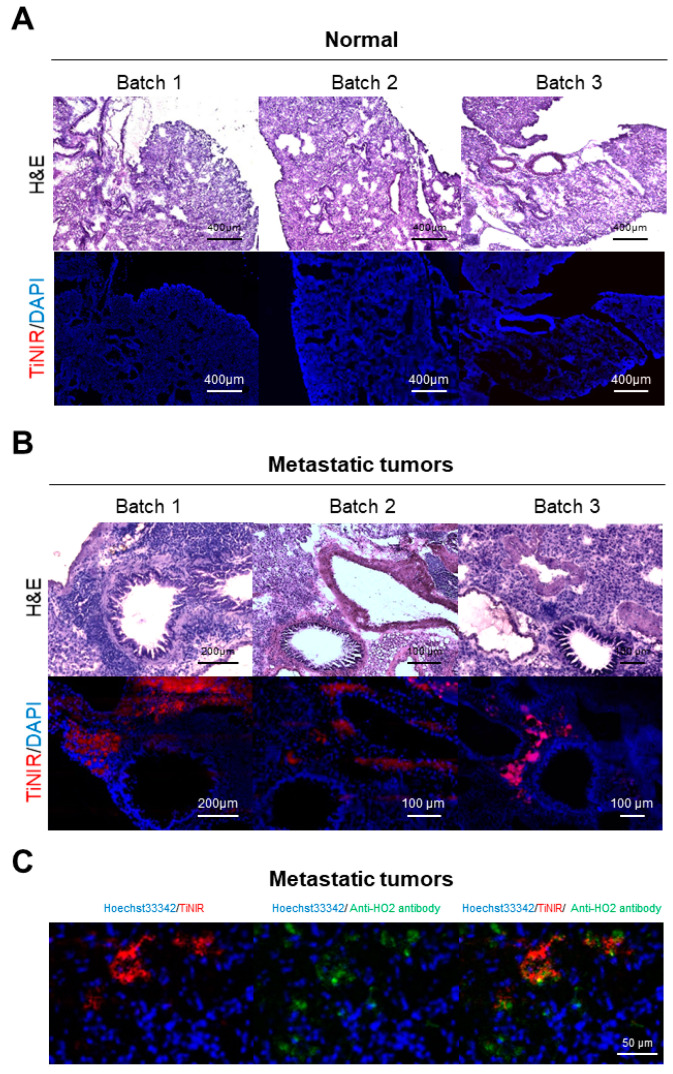
Histological analysis of the lungs from the TiNIR-injected normal group (**A**) and the metastatic cancer mouse (**B**). The lung tissues were stained by H&E to confirm the generation of the tumor (upper). (**C**) Confirming co-localization of the TiNIR signal with the HO2 antibody. The TiNIR-injected mouse lung tissue was stained by an HO2 antibody (Alexa-488). Images were analyzed by EVOS 7000 microscope with a ×20 (H&E) and ×40 (fluorescence) air objective.

The validation of metastatic cancer generation was confirmed through histological analysis via H&E staining. The lungs of mice induced with metastasis observed tumor areas, as opposed to normal lungs (as observed in Figure 4A). The TiNIR signal was detected in similar tumor areas, as seen in the H&E stain pattern (as shown in Figure 4B). To confirm the co-localization of TiNIR and HO2 protein, we treated the HO2 antibody. The TiNIR signal was located in a similar area to the HO2 antibody signal (Figure 4C). By establishing this realistic metastatic cancer model, we were able to mimic actual cancer treatment processes, as well as demonstrate the selectivity and potential for visualizing TiNIR for metastatic cancer in *ex vivo* and tissue imaging. This not only provides valuable insights for the detection and tracking of metastatic cancer but also serves as a promising tool for evaluating the efficacy of cancer treatments.

## 4. Discussion

Detecting metastatic cancer at an early stage is crucial for successful treatment outcomes [22]. Researchers are exploring new approaches, such as probes and new biomarkers. Therefore, the development of an effective biomarker and its probe for tracking and treating cancer, especially metastatic cancer is one of the important factors for maintaining good quality of life for patients, and it also provides a new experimental tool to examine drug discovery [8,9,10].

We previously developed an HO2 tracker TiNIR to treat and track lung TICs [18]. TICs are responsible for the generation of cancer and resistance to drugs/radiotherapy [23,24,25,26]. TICs are known to respond more rapidly to DNA damage by activating DNA repair proteins compared to normal cells and differentiated tumor populations [17]. TICs exhibit high expression of drug-resistance transporters such as ABCB1, the ABCC family, and ABCG2, which contributes to their ability to resist cytotoxic compounds [27]. Moreover, TICs show significant upregulation of aldehyde dehydrogenase 1 (ALDH1), which has been strongly associated with tumor resistance to treatment [28]. Because of these reasons, monitoring and controlling TICs through TiNIR gives benefits for the early diagnosis and treatment of patients [18].

Here, we expand the applicability of TiNIR to metastatic cancer. HO2 has emerged as a therapeutic biomarker for TICs and other cancers. Thus, before utilizing TiNIR, it is important to validate the expression levels of HO2 (heme oxygenase 2) in different stages of cancer cells. In this study, we focused on comparing the expression levels of HO2 in stationary cancer cells versus actively migrating cancer cells. We verified the increased expression level of HO2 in the metastatic cancer cells *in vitro* and *in vivo*. It reflects the possibility of the usability of TiNIR; *in vivo* application clearly confirmed TiNIR as a metastatic lung cancer tracker. TiNIR and its binding partner, HO2, hold potential in cancer research and therapy. Further studies are necessary to fully explore the therapeutic potential of these probes and their role in metastasis. The development of innovative approaches that selectively target TICs may ultimately improve survival rates and quality of life for cancer patients.

To track tumors in cancer studies, researchers often use genetically modified strategies such as GFP, RFP, and luciferase assays [29,30]. These systems can efficiently utilize animal models to explore oncology and drug discovery. However, genetically engineered animal models have limitations, such as the need for gene manipulation or expensive animal models, and genetic modifications can sometimes alter the characteristics of cancer [31]. Therefore, we want to emphasize that TiNIR can provide an easy way to visualize metastatic cancer using realistic animal models.

## Data Availability

Not applicable.
